# Host mitochondria: more than an organelle in SARS-CoV-2 infection

**DOI:** 10.3389/fcimb.2023.1228275

**Published:** 2023-08-25

**Authors:** Shahrzad Shoraka, Amali E. Samarasinghe, Amir Ghaemi, Seyed Reza Mohebbi

**Affiliations:** ^1^ Basic and Molecular Epidemiology of Gastrointestinal Disorders Research Center, Research Institute for Gastroenterology and Liver Diseases, Shahid Beheshti University of Medical Sciences, Tehran, Iran; ^2^ Department of Microbiology and Microbial Biotechnology, Faculty of Life Sciences and Biotechnology, Shahid Beheshti University, Tehran, Iran; ^3^ Division of Pulmonology, Allergy and Immunology, Department of Pediatrics, College of Medicine, University of Tennessee Health Science Center, Memphis, TN, United States; ^4^ Children’s Foundation Research Institute, Memphis, TN, United States; ^5^ Department of Virology, Pasteur Institute of Iran, Tehran, Iran; ^6^ Gastroenterology and Liver Diseases Research Center, Research Institute for Gastroenterology and Liver Diseases, Shahid Beheshti University of Medical Sciences, Tehran, Iran

**Keywords:** coronavirus, SARS-CoV-2, COVID-19, mitochondria, viral infection

## Abstract

Since December 2019, the world has been facing viral pandemic called COVID-19 (Coronavirus disease 2019) caused by a new beta-coronavirus named severe acute respiratory syndrome coronavirus-2, or SARS-CoV-2. COVID-19 patients may present with a wide range of symptoms, from asymptomatic to requiring intensive care support. The severe form of COVID-19 is often marked by an altered immune response and cytokine storm. Advanced age, age-related and underlying diseases, including metabolic syndromes, appear to contribute to increased COVID-19 severity and mortality suggesting a role for mitochondria in disease pathogenesis. Furthermore, since the immune system is associated with mitochondria and its damage-related molecular patterns (mtDAMPs), the host mitochondrial system may play an important role during viral infections. Viruses have evolved to modulate the immune system and mitochondrial function for survival and proliferation, which in turn could lead to cellular stress and contribute to disease progression. Recent studies have focused on the possible roles of mitochondria in SARS-CoV-2 infection. It has been suggested that mitochondrial hijacking by SARS-CoV-2 could be a key factor in COVID-19 pathogenesis. In this review, we discuss the roles of mitochondria in viral infections including SARS-CoV-2 infection based on past and present knowledge. Paying attention to the role of mitochondria in SARS-CoV-2 infection will help to better understand the pathophysiology of COVID-19 and to achieve effective methods of prevention, diagnosis, and treatment.

## Highlights

- Mitochondria are the “powerhouse” of the cell and have several other important functions. Multiple mitochondrial functions make them essential to the cell, so when a virus “hijacks” mitochondrial function, this allows it to control the entire cell.
- Studies have suggested that host mitochondria are hijacked by the SARS-CoV-2 based on *in silico* predictions. Emerging evidence also suggested that SARS-CoV-2 hijacks the mitochondria of immune cells, proliferate in mitochondrial structures, and impairs mitochondrial functions. The data indicate that patients with COVID-19 have a population of T cells with mitochondrial dysfunction, as well as mitochondrial markers in monocytes have altered.
- The interaction of several SARS-CoV-2 proteins with mitochondrial components components may occur.
- Lung tissue studies in COVID-19 patients have shown that mitochondrial function is altered following infection.
- Monitoring mitochondrial function of immune cells in the blood could be a non-invasive marker to diagnose and predict the prognosis of COVID-19.
- Understanding the cause of more severity and mortality in people with underlying diseases, particularly metabolic and age-related diseases, could help improve the management of the COVID-19 pandemic.
- There are various strategies with pharmacological agents and lifestyle interventions to target mitochondria that could be promising to prevent SARS-CoV-2 infection or reduce disease progression. However, there is little empirical evidence of their potential.
- Given the important roles of mitochondria in viral infection, mitochondria could be considered a therapeutic target for COVID-19 disease.

## Introduction

The new member of the coronavirus family, like other members, is enveloped virus containing a positive single-stranded RNA [(+) ssRNA] genome. During the last two decades, and before the current COVID-19 pandemic, coronaviruses also caused two epidemic diseases, SARS (severe acute respiratory syndrome) in 2003 and MERS (the Middle East respiratory syndrome) in 2012. SARS-CoV-2 like the SARS-CoV and MERS-CoV, belongs to the beta group of coronaviruses and, similar to SARS-CoV, belongs to the B lineage of betacoronaviruses (Eriani and Martin, 2022). The SARS-CoV-2 genome encodes 4 structural proteins (spike (S), envelope (E), membrane (M), nucleocapsid protein (N)) and 16 nonstructural proteins (NSP1-16) that are important for replication, infection, and triggering host immune response (Bai et al., 2022).

SARS-CoV-2 invades the host cell by attaching its surface spike protein to cell receptors such as angiotensin-converting enzyme 2 (ACE2), a receptor expressed in a variety of organs (Kuppusamy et al., 2021). Most COVID-19 patients present with respiratory symptoms, although clinical manifestations of extra-respiratory symptoms have also been reported. Also, COVID-19 patients may show a wide range of symptoms, from asymptomatic to severe disease (Shoraka et al., 2021). Although the main cause of severe COVID-19 is not clearly understood, it appears to correlate with host immune-viral interactions, excessive and/or impaired inflammatory response and cytokine storm play important roles in this regard (Zheng and Gao, 2023).

### Mitochondria

The mitochondrion is an organelle in the cytoplasm that function as the energy wheelhouse of eukaryotic cells. Mitochondrial DNA (mtDNA) is a 16.5 kb extra-chromosomal circular double-stranded DNA. The number of mtDNA copies in a cell varies depending on the cell type. mtDNA copy number could also change in response to physiological signals and some diseases (Hummel et al., 2023).

Mitochondrial perform numerous other cellular functions in addition to cell energy production by performing oxidative phosphorylation (OXPHOS), serving as the main site of reactive oxygen species (ROS) generation as a byproduct of the electron transfer chain (ETC), and controlling cellular processes involved in metabolism and immune responses (Kuznetsov et al., 2022).

### Role of mitochondria in viral infection

The prominent role of mitochondria in the outcome of viral infection has been demonstrated in the clinical phenotype of patients with inherited mitochondrial defects (Kruk et al., 2019). In 2015, Thakar et al. reported that decreased efficacy of the influenza vaccine is associated with decreased mitochondrial biogenesis (Thakar et al., 2015). Mitochondrial dysfunction has been also considered not only in HIV infection but also in antiviral drug therapies (Ganta and Chaubey, 2019). Thus, impaired mitochondrial function could affect both the host response to the virus and the development of an effective response to vaccine or treatment (Nunn et al., 2020).

The role of mitochondria in the immune system of mammals has been substantially proven. Many studies show that mitochondria and mitochondrial damage-related molecular patterns (mtDAMPs), such as mtDNA, cardiolipin, N-formyl peptides, and cytochrome c, have an important role in the host immune response against viral infections (Garg et al., 2022). Since the host immune response against viruses is significantly based on mitochondrial function, it is not surprising that viruses modulate mitochondrial function to promote their proliferation and survival (Tiku et al., 2020). In the following section, mitochondrial roles in viral infection will be discussed briefly.

### Mitochondrial antiviral signaling (MAVS)

The innate immune response is the first line of host defense against viruses and begins with the identification of pathogen-related molecular patterns (PAMPs) or DAMPs by pattern recognition receptors (PRRs). This leads to activation of some signaling pathways and subsequently, induction of interferons (IFNs), pro-inflammatory cytokines, and other antiviral effector genes. Among PRRs, RIG-I-like receptors (RLRs) and MDA5 have been identified as cytosolic viral RNA sensors in mammalian cells. After viral RNA binding, RLRs translocate to mitochondrial adaptor protein MAVS (mitochondrial antiviral signaling) in the mitochondrial membrane and activate MAVS to induce the formation of oligomers in a prion-like manner. The formation of the MAVS complex is modulated by mitochondrial translocase of outer membrane 70 (Tom70) and 20 (Tom20). This pathway can either activate type I IFN transcription and stimulate interferon-stimulated genes (ISGs) or release NF-κB to activate the transcription of pro-inflammatory cytokines. While both pathways converge on antiviral immunity, overstimulation of the second pathway may lead to cytokine storms and viral persistence in infected cells (Fu et al., 2021; Sharma et al., 2021). During RNA virus infection, MAVS also appears to play a major role in activating NLRP3 inflammasome. Inflammasome activation often leads to programmed pro-inflammatory cell death known as pyroptosis with heightened secretion of interleukin-1β (IL-1β) (Paik et al., 2021).

The MAVS pathway is targeted by many viruses. Some DNA or RNA viruses have evolved mechanisms targeting mitochondria to escape IFN-I-mediated host immune responses (Iessi et al., 2020). Human Rhinovirus C encodes a protease that could cleave MAVS to inhibit RLR signaling (Pang et al., 2017). The influenza virus protein PB1-F2 binds to and disrupts MAVS and reduces the IFN response (Varga et al., 2012). Similarly, the ORF-9b protein in SARS-CoV restricts host IFN responses by degrading MAVS. ORF-9b leads to viral escape by manipulating mitochondrial function (Shi et al., 2014). Also, SARS-CoV ORF-7a and ORF-8b can translocate to mitochondria and alter MAVS and mitochondrial function (Ganji and Reddy, 2021). These data suggest that blocking innate immune signaling through mitochondrial manipulation could be one of the viral evasion strategies (Ferreira et al., 2016).

Studies have demonstrated that SARS-CoV-2 RNA could activate RIG-I/MAVS signaling pathway (Wu et al., 2021). Downregulation of MAVS has also been confirmed in SARS-CoV-2-infected CaCo-2 (human colon epithelial carcinoma) cells (Bojkova et al., 2020). Studies suggest that SARS-CoV-2 uses several conserved virulence genes to antagonize the IFN response (Wu et al., 2021; Znaidia et al., 2022) (See **Table 1**).

**Table 1:** Interaction of SARS-CoV-2 proteins with host immune response components and modulation of MAVS signaling.

SARS-CoV-2 protein	Mechanisms	Reference
**NSP1**	Blocks RIG-I-dependent innate immune responses by associating with ribosomes to inhibit translation of RIG-I and ISGs	(Thoms et al., 2020)
**NSP5**	Targets RIG-I and MAVS protein through two distinct mechanisms to escape the host innate immune response	(Liu et al., 2021b)
**NSP7**	Reduction in type I and III IFN responses through disruption of RIG-I/MDA5-MAVS, TLR3-TRIF, and cGAS-STING signaling pathways	(Deng et al., 2023b)
**NSP8**	By disrupting the formation of the RIG-I/MDA5-MAVS complex and targeting TRIF and STING signaling, it leads to the suppression of type I and III IFN responses	(Deng et al., 2023a)
**NSP13**	Interacts with key intermediates in IFN signaling pathway, namely TBK1, TBKBP1	(O’Meara et al., 2020)
**NSP15**	Interacts with key intermediates in IFN signaling pathway, namely TBK1, RNF41	(O’Meara et al., 2020)
**ORF-3c**	Suppression of RIG-I- and MDA5-mediated immune activation and interaction with MAVS and subsequently inhibition of IFN-β induction	(Mueller et al., 2023)
**ORF-9b**	Inhibits the activation of IFN-I and -III by targeting different molecules of the innate immune signaling pathway. Furthermore, ORF-9b can inhibit RIG-I/MDA5-MAVS signaling pathways through interaction with the mitochondrial surface receptor, Tom70	(Jiang et al., 2020; Han et al., 2021)
**ORF-10**	Induces mitophagy-mediated degradation of MAVS by binding to Nip3-like protein X (NIX)	(Li et al., 2022)
**Membrane glycoprotein M**	Serves as a negative regulator of the innate immune response by interacting with MAVS protein which disrupts the accumulation of MAVS and leads to attenuation of the innate antiviral response	(Fu et al., 2021)
**Nucleocapsid protein (NP)**	Suppresses the type I IFN response through direct interaction with the MAVS protein	(Zotta et al., 2021)

### Mitochondrial dynamics

Mitochondria are also dynamic organelles that modulate and adapt in response to environmental stresses. Mitochondrial dynamics include fusion and fission events, mitochondrial autophagy (mitophagy), and biogenesis. DRP1 and FIS1 proteins play key roles in mitochondrial fission (a process by which mitochondria divide and are essential for cell growth and division) and sometimes occur during mitochondrial damage. Fission increases ROS production and also facilitates mitophagy and mitochondrial distribution. Mitochondrial fusion, wherein they elongate, maximizes oxidative capacity in response to stress. MFN1, MFN2, and OPA1 proteins are involved in the fusion process (Giacomello et al., 2020; Al Ojaimi et al., 2022). In contrast to fission that occurs during nutrient-replete states resulting in nutrient storage and reduced bioenergetics efficiency, mitochondrial fusion is promoted in nutrient-deficient conditions and increases the bioenergetics efficiency of mitochondria (Schrepfer and Scorrano, 2016; Giacomello et al., 2020).

Changes in metabolic or physiological conditions, such as infection, induce mitochondrial morphological alterations that have functional consequences which significantly affect the innate immune response (Banoth and Cassel, 2018). The close association between RLR signaling and mitochondrial dynamics has been confirmed. Efficient RLR signaling requires MAVS interaction with MFN1, while mitochondrial fission factor (MFF1) or DRP1 as a fusion inhibitor reduces virus-induced NF-κB and interferon regulatory transcription factor 3 (IRF-3) activation. After depletion of DRP1 and FIS1 in cells, RLR signaling increases, and elongation of the mitochondrial network enhances the endoplasmic reticulum (ER)-mitochondria interaction during viral infection and increases the association of MAVS to enhance RLR signaling (Castanier et al., 2010; Kim et al., 2018).

Therefore, the lack of the *drp1* gene leads to improved antiviral responses during infection through mitochondrial elongation, while inhibition of MFN1 and OPA1 leads to mitochondrial degradation and decreased antiviral response (Kim et al., 2018; Lai et al., 2018). Also, *drp1* knockdown targets NLRP3 inflammasome accumulation and activates caspase-1 and IL-1β (Park et al., 2015).

Viruses are able to manipulate the function of cellular factors affecting mitochondrial dynamics such as DRP1, MFN, and OPA1 pushing mitochondria toward fusion or fission. Viral infections may lead to mitochondrial elongation, potentially increasing antiviral signals (Castanier et al., 2010). Also, DRP1 is usually targeted by viruses. For example, HIV protein gp120 could impair mitochondrial fission. Mitochondrial fission is important for clearance of defective mitochondria (Fields et al., 2016). Conversely, some viruses, such as hepatitis B virus, alter mitochondrial dynamics toward fission and mitophagy thus leading to viral persistence (Kim et al., 2013). The ORF-9b protein of SARS-CoV in addition to degrading MAVS, could alter mitochondrial dynamics and modulate host IFN responses. ORF-9b protein causes DRP1 degradation and mitochondrial elongation through physical interaction with DRP1 (Shi et al., 2014).

Studies of lung tissue in patients with COVID-19 show that the mitochondrial dynamic balance is disturbed, thus resulting in oxidative stress, pro-inflammatory status, cytokine production, and cell death (Tay et al., 2020). An early study on SARS-CoV-2 infection proposed an increase in fusion, leading to mitochondrial elongation, preventing apoptosis, and creating an intracellular environment suitable for virus propagation in infected cells (Holder and Reddy, 2021). A recent study also showed that mitochondria were strongly fused in SARS-CoV-2 positive human placenta (Gabanella et al., 2022).

### Mitochondrial DNA

Mitochondrial DNA damage increases the mtDNA copy numbers, seemingly as a compensatory mechanism. The number of mtDNA copies is a reflection of the loss of mtDNA integrity and a marker for mitochondrial function and oxidative stress (Riou et al., 2020).

In addition, the ratio of the mitochondrial genome to the nuclear genome (mt/n) may change during oxidative stress. The initial response to increased oxidative stress could be an adaptive response in which the mt/n ratio increases as a result of heightened mitochondrial biogenesis. While prolonged oxidative stress may lead to a decrease in mt/n ratio with mitochondrial dysfunction due to damage to mitochondrial DNA and proteins. The inflammatory response could be triggered by the accumulation of damaged mtDNA in the cell (Malik and Czajka, 2013). In some conditions, mtDNA could leak into the cytoplasm and extracellular space triggering inflammation. Various factors could contribute to mtDNA release, for example, the stress induced by the absence of TFAM (a transcription factor that binds to mtDNA) leads to improper packaging of mtDNA and ultimately its release into cytosol (Rongvaux, 2018). Some immune pathways, such as IL-1β signaling, leading to mitochondrial stress and mtDNA release (Cheng et al., 2020). In addition, many viruses can cause mitochondrial stress. As reported for Herpesvirus 1 (HSV-1), cell-free mtDNA (cf-mtDNA) targets inflammatory and antiviral responses. This signaling mechanism is conserved in many viral infections (Corcoran et al., 2009). Metabolic stress due to HIV replication has a role in mitochondrial damage and mtDNA leakage (Pernas et al., 2017). Studies showed that positive single-stranded RNA viruses produce a strong inflammatory response that involves the release of mtDNA out of the cell (Storci et al., 2020a). Also in a process termed NETosis (neutrophil extracellular trap), neutrophils release their nuclear and mitochondrial DNA into the surrounding tissue when stimulated by virus infection (Cao et al., 2022).

In addition, mtDNA is a circular loop that contains a significant number of CpG islands evolved from prokaryotic symbiosis. Therefore extracellular mtDNA could target various pro-inflammatory signaling pathways (Comish et al., 2022). Toll-like receptor-9 (TLR-9) induces NF-κB-dependent pro-inflammatory signaling by binding to mtDNA. Also, the detection of mtDNA in the cytosol by cGAS involves activation of stimulator of interferon genes (STING) (located in the endoplasmic reticulum) and induction of IFN response. The activated cGAS-STING pathway also induces NF-κB pro-inflammatory signaling. Activation of mtDNA-dependent inflammasome also leads to the production of pro-inflammatory IL-1 and IL-8 (Riley and Tait, 2020).

In recent studies, a significant association between serum/plasma mtDNA levels and various diseases such as trauma or viral and bacterial infections has been demonstrated (Cossarizza et al., 2011). These findings suggest that cell-free mtDNA is a marker for cell death and pathogen-induced disease severity (Gambardella et al., 2019). The presence of cell-free mtDNA in the plasma of COVID-19 patients has been reported, and there is a significant relationship between the high level of mtDNA and the disease severity (Edinger et al., 2022; Leonard et al., 2022). However, the source and mechanism of releasing mtDNA in response to SARS-CoV-2 needs further investigation. The ability of mtDNA to activate TLR-9, cGAS and inflammasomes has made circulating mtDNA a candidate for the pro-inflammatory response in COVID-19 patients, especially in the elderly (Storci et al., 2020b).

In addition to mitochondrial DNA, other mitochondrial DAMPs have also been investigated in COVID-19 (Garg et al., 2022). Mitochondrial N-formylmethionine peptides contribute to neutrophil hyperactivation in COVID-19 patients (Kuley et al., 2023). Higher production of anti-cardiolipin antibodies in patients with COVID-19 may also be related to DAMP activity of mitochondrial membrane cardiolipin (Bhowal et al., 2023). Although studies on mitochondrial RNAs such as mitochondrial long non-coding RNAs (mt-lncRNAs) and mitochondrial small RNAs (mt-sRNAs) are limited, it has been suggested that double-stranded mitochondrial RNAs (mtRNA) triggers antiviral signaling. Cytosolic mitochondrial RNA is recognized by RIG-I or MDA5 and ultimately leads to immune activation. A recent study showed that the expression of mt-sRNAs may change during COVID-19 recovery, suggesting these relatively unknown RNAs may be good candidates for further studies (Pozzi, 2022; Marchi et al., 2023).

### Activation of the cGAS-STING pathway by released mtDNA

**Figure 1:**
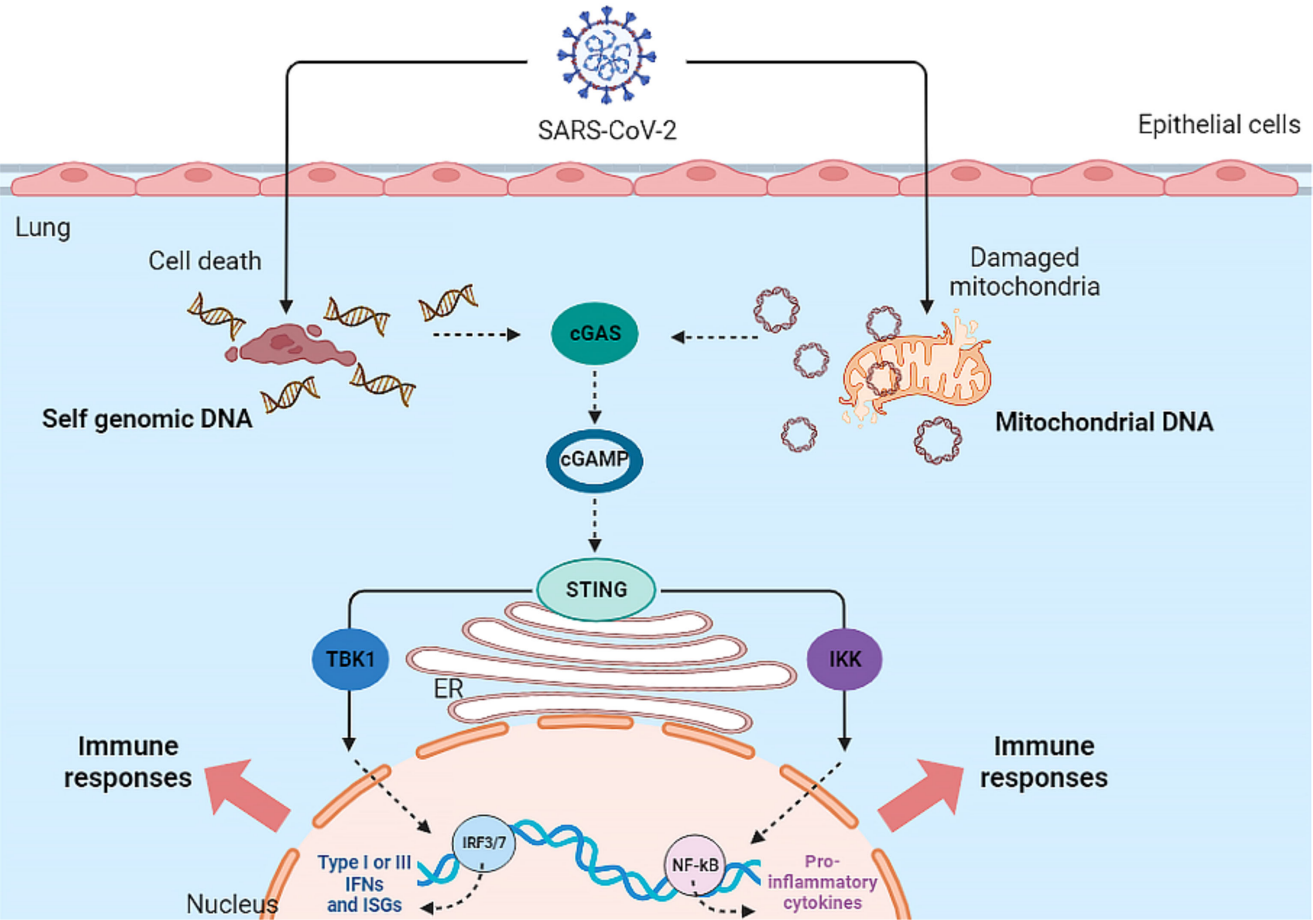
Activation of cGAS-STING Pathway by SARS-CoV-2 infection. The SARS-CoV-2 virus indirectly activates the cGAS-STING pathway and ultimately the inflammatory response in the host cell.

In addition to the MAVS pathway, mtDNA -since it acts as a DAMP- could also be involved in the immune response. As mentioned, released mtDNA could activate both the NF-κB signaling pathway and the cGAS-STING pathway. Therefore, cell-free mtDNA can activate innate immune signaling pathways (Mills et al., 2017) (See **Figure 1**).

The cGAS-STING pathway is activated by mtDNA in various cells such as macrophages, dendritic cells (DCs), endothelia, and epithelia, resulting in the production of type I IFN and the release of inflammatory cytokines. In addition, the cGAS-STING signaling pathway interacts with apoptosis, necrosis, pyroptosis, and also autophagy (Ma et al., 2020). To inhibit or limit cGAS activation, viruses use various strategies to destroy or reduce cGAS. Dengue virus NS2B3 protease cleaves cGAS in order to suppress its activation (Bhattacharya et al., 2023). Papain-like protease (PL-pro) in HCoV-NL63 coronavirus disrupts STING oligomerization and ultimately leads to virus escape from the innate immune system (Sun et al., 2012). Manipulation of the cGAS-STING pathway by PLpro has also been reported in SARS-CoV, resulting in the regulation of the host NF-κB and IFN pathways (Ma and Damania, 2016).

Recent studies have attributed a delayed cGAS-STING response to SARS-CoV-2 infection. At the onset of SARS-CoV-2 infection, the virus ORF-9b protein inhibits the induction of type I and III IFNs by inhibiting the cGAS-STING signaling pathway. ORF-9b exerts its inhibitory effect by interacting with STING and inhibiting TBK1 phosphorylation (Han et al., 2021). Studies have shown that other SARS-CoV-2 proteins, ORF-3a, ORF-10, NSP7, NSP8, PLpro and 3CL protease, can also inhibit the STING pathway and the subsequent interferon response, leading to viral escape from innate immunity (Rui et al., 2021; Han et al., 2022; Deng et al., 2023b; Deng et al., 2023a). However, in the secondary phase of COVID-19, damaged host DNA could lead to STING over-activation, release of IFN-β, and cytokine storm following IRF-3 and NF-κB activation (Berthelot and Lioté, 2020).

### Mitochondria-induced inflammasome activation

Inflammasomes are macromolecular immune complexes that are activated in response to PAMPs and DAMPs and lead to the activation, maturation, and release of pro-inflammatory cytokines. Inflammasomes play a dual role in viral infection. Activated inflammasomes provide inflammatory caspases to induce pyroptosis and cytokine production, leading to the elimination of virus-infected cells. However, impaired inflammasome activation could lead to a cytokine storm and hyperinflammation (Wang et al., 2023). Several molecular and cellular events have been shown to activate inflammasomes. Many studies have suggested the role of ROS, mitochondrial dysfunction, and mtDNA in NLRP3 inflammasome activation (Kelley et al., 2019). MAVS also physically interacts with the NLRP3, and it has been suggested that MAVS is required for the NLRP3 inflammasome activation by viral infections (Franchi et al., 2014). NLRP3 inflammasome activity is negatively regulated by autophagy (Zhou et al., 2011). In addition, free DNA or mtDNA can activate the AIM2 and NLRP3 inflammasomes (Uresti-Rivera and García-Hernández, 2023).

Aberrant inflammasome activation has been reported in COVID-19 patients (Aymonnier et al., 2022). SARS-CoV-2 activates inflammasomes through several pathways. SARS-CoV-2 infection can lead to increased expression and synthesis of the NLRP3 and IL-1β by upregulating the NF-κB pathway. SARS-CoV-2 proteins including S, E, and NSP2 stimulate NLRP3 transcription Also, viral N protein directly interacts with NLRP3 to induce inflammasome formation and subsequent inflammatory response. On the other hand, the inflammasomes can be activated in a way related to dsDNA released from infected epithelial cells and mtDNA released from damaged mitochondria. Mitochondrial ROS and lysosomal degradation further activate the NLRP3 inflammasome (Dutta et al., 2022; Deng C-H. et al., 2023).

### Mitochondrial apoptosis pathway

Cellular apoptosis is a final resort in defense against invading pathogens. Programmed cell death eliminates the infected cell and limits the production of viruses. In addition, activation of antiviral transcription and cell death are necessary to control viral infection and provide long-term immunity (Orzalli and Kagan, 2017). The three main pathways for activating the caspase and apoptosis signals in mammalian cells include; the extrinsic pathway, intrinsic pathway (mitochondrial pathway), and cell death mediated by granzyme (Murthy et al., 2020). Mitochondria are directly and indirectly involved in apoptosis (Ghosh and Sil, 2021).

The intrinsic pathway is activated in response to cellular stress and requires mitochondrial outer membrane permeability. This permeability is induced by BAX/BAK proteins which are in the cytoplasm inactivated by BCL-2 protein at a steady state. In response to apoptotic stimulation, BAX/BAK is transported into the mitochondria and undergoes conformational changes, releasing cytochrome c from mitochondria and activation of downstream caspases. Caspase-9 is activated and leads to cell death via cleavage of caspase-3 and caspase-7 (Orzalli and Kagan, 2017).

Disruption of mitochondrial fusion/fission machinery can also lead to mitochondrial destruction and the initiation of apoptotic processes (Scaini et al., 2017). Loss of mitochondrial membrane potential (MMP, Δψm) leads to an imbalance between outer and inner membrane potentials, arresting normal cellular biosynthetic functions and bioenergetics within the cell. MMP changes occur during the pathogenesis of viral proteins, toxins, and pro-oxidants. Long-term loss of MMP leads to serious irreparable cell damage. Therefore, any viral agent that affects MMP has a major effect on cellular fate, either by inducing or inhibiting cell death (Reshi et al., 2014).

On the other hand, mitochondrial apoptosis could inhibit inflammatory signaling initiated by mtDNA. The role of mtDNA in initiating cGAS-STING mediated IFN response and inhibition of this inflammatory response by activation of caspase-9 has been demonstrated. Mitochondria have a dual capacity; on the one hand, using mtDNA to induce an IFN response, and on the other hand, with the help of caspase, it inactivates this response (Murthy et al., 2020). The mechanism of reducing the inflammatory response by the cascade of apoptotic caspases has recently been identified. Caspase-3 prevents the overproduction of type I IFN during viral infection by cleavage of cGAS, IRF-3, and MAVS. By inhibiting caspases, mitochondrial permeability leads to activation of cGAS-STING and transcription of NF-κB. The cytokines induced by NF-κB could lead to macrophage activation (Murthy et al., 2020; Riley and Tait, 2020). Viruses utilize a variety of strategies to prevent apoptosis; such as inducing the expression of survival genes such as anti-apoptotic BCL-2 proteins. (+)ssRNA viruses appear to activate mitophagy to remove damaged mitochondria and thus prevent apoptosis. SARS-CoV has increased the survival of infected cells by inhibiting apoptosis through mitochondrial fusion (Prasun, 2020). Conversely, SARS-CoV 3a protein could activate mitochondrial cell death pathways (Padhan et al., 2008). Also, MERS-CoV effectively activates extrinsic and intrinsic apoptotic pathways in T cells (Chu et al., 2016).

SARS-CoV-2 infection in lung epithelial cells activates caspase-8 and also targets apoptosis and the processing of inflammatory cytokines such as IL-1β into the active form (Li et al., 2020). SARS-CoV-2 ORF-3a and ORF-7b induces apoptosis *in vitro* (Ren et al., 2020; Yang et al., 2021). A recent study demonstrated that SARS-CoV-2 could induce both extrinsic and intrinsic apoptotic pathways (Liu et al., 2021a).

### Autophagy and mitophagy

Autophagy is responsible for the selective removal of dysfunctional organelles, intracellular pathogens, and misplaced proteins, as well as the regulation of the immune response. The autophagy process is induced by *Atg* genes and is regulated by several signaling pathways such as AMPK, MAPK/ERK, and PI3K/Akt. mTOR is a negative regulator of autophagy. The dual role of autophagy in the formation and regulation of NETs has been proven. Thus autophagy is closely related to the host inflammatory response. Autophagy has a protective role in reducing the excessive release of cytokines in ARDS (Cheng et al., 2020; Cicco et al., 2020).

During viral infections, TLR activation induces autophagy and enhances IFN production, while negative regulation of autophagy helps terminate TLR signaling. Studies also show the induction of STING-dependent autophagy during viral infections. Some coronaviruses negatively regulate the IFN response through autophagic degradation of key molecules in the IFN signaling cascade. Manipulation of the autophagy process by various viruses has been reported (García-Pérez et al., 2020). SARS-CoV ORF-9b protein could induce autophagy and activate NF-κB (Shi et al., 2014).

The selective destruction of mitochondria by the process of autophagy is called mitophagy. Under normal conditions, damaged mitochondria are removed from the cell by mitophagy. The proteins involved in this pathway are PINK1 and PARKIN. Studies confirm that mice without the *parkin* or *pink1* genes show higher levels of pro-inflammatory IFN-β and IL-6 than wild-type mice. An increase in cell-free mtDNA has also been observed in mice without *parkin* (Riley and Tait, 2020).

Mitophagy is also manipulated by various factors including viral infections. Some viruses directly or indirectly control the mitophagy process with different strategies. This promotes infection and reduces the host innate immune responses. Viral infections inhibit inflammasome activation by inducing mitophagy. In the absence of mitophagy, mitochondrial accumulation could increase MAVS levels and thus enhance its antiviral signaling pathway. Therefore, mitophagy induced by viral infection leads to decreased MAVS signaling and IFN-I response (Chen T. et al., 2023). (+)ssRNA viruses could activate mitophagy to remove damaged mitochondria and prevent apoptosis. SARS-CoV targets mitophagy with the help of ORF-9b protein (Gatti et al., 2020).

SARS-CoV-2 NSP8 induces mitophagy and incomplete autophagy *in vivo* (Zong et al., 2023). SARS-CoV-2 ORF-3c impairs autophagy *in vivo* (Mozzi et al., 2023). Also, SARS-CoV-2 ORF-7a initiates autophagy and restricts autophagosome-lysosome fusion to promote virus replication (Hou et al., 2023). A 2023 study showed that both S1 and RBD proteins, by inhibiting mitophagy and increasing mtROS, lead to IL-18 expression and inflammasome NLRP3 activation *in vivo* and *in vitro* (Liang et al., 2023).

### ROS and antioxidant defense system

ROS are produced during mitochondrial oxidative metabolism or in response to ultraviolet (UV) radiation, xenobiotics, bacterial invasion, and viral infection. Mitochondria are thought to play a major role in the generation of intracellular ROS in most cell types. ROS participates in cellular signaling as secondary messengers and could regulate hormone action, cytokines, apoptosis, and immunomodulation (Reshi et al., 2014; El-Amine et al., 2018). ROS have a role in innate immunity as defense mechanism as well as in cell types involved in the acquired immune response (Yang et al., 2013).

Oxidative damage to DNA, proteins, and lipids is associated with increased ROS production, mitochondrial dysfunction, and eventually aging and cell death. Continuous production of ROS by mitochondria throughout cell life causes age-related chronic oxidative stress which leads to oxidative modification or deletion of bases especially in mtDNA (Giorgi et al., 2018). Overproduction of ROS and deficiency in antioxidant defense systems lead to oxidative stress, a condition that may contribute to various human diseases and is common during viral invasion (Reshi et al., 2018).

The main target of ROS which are produced inside a cell during viral infection is the mtDNA. Because mitochondrial ATP production requires proteins from the nuclear and mitochondrial genomes, damage to the mtDNA by ROS disrupts oxidative ATP production, although ROS have other cellular targets (Shokolenko et al., 2009; Reshi et al., 2018). Oxidative stress caused by the virus through the production of ROS is important for the life cycle and pathogenicity of the virus (Bermano et al., 2021). Oxidative stress could reduce the function of the host immune system. Almost all viruses including RNA viruses cause cell death by producing oxidative stress in infected cells. In hepatitis C virus infection and HIV, oxidative stress always plays a significant role in pathogenesis (Couret and Chang, 2016; Ivanov et al., 2018; Reshi et al., 2018). Respiratory viral infections are associated with inhibition of Nrf2 antioxidant pathways and/or activation of NF-κB signaling, leading to inflammation and oxidative stress. Several respiratory viruses cause the formation of dysregulated ROS as a result of increased inflammatory cells at the site of infection. Furthermore, viral infections may disrupt antioxidant mechanisms, leading to an imbalanced status and consequent oxidative cell damage (Komaravelli and Casola, 2014). Several studies suggested that the onset of severe lung injury in SARS-CoV patients depends on the activation of the oxidative stress system, which is coupled with innate immunity and activates transcription factors such as NF-κB, leading to an intensified pro-inflammatory host response (Delgado-Roche and Mesta, 2020). Lin et al. showed that SARS-CoV 3CLpro caused a significant increase in ROS production in HL-CZ cells. They also suggested that the ROS-activated NF-κB signaling pathway, induced by SARS-CoV 3CLpro, may be a key player in the pathophysiology of SARS-CoV (Lin et al., 2006).

Biopsy of COVID-19 patients demonstrated decreased expression of genes related to the Nrf2 pathway, which may be associated with viral oxidative cell damage (Olagnier et al., 2020). Also, a recent study demonstrated that serum ROS levels were significantly higher in COVID-19 patients compared to healthy controls and in ICU patients compared to non-ICU patients (Ahmed et al., 2023). The high neutrophil-to-lymphocyte ratio described in the severe form of COVID-19 is associated with excessive levels of ROS. This increases the cascade of biological events that drive the pathological host response (Laforge et al., 2020). A recent study reported that the release of TNF-α during a cytokine storm intensified ROS production through a positive feedback loop. Production of TNF-α-mediated ROS may be an important contributing factor to the observed damage to distal tissue such as the brain in COVID-19 patients (Wichmann et al., 2020). ROS storms may also occur in response to and/or lead to the breakdown of the antioxidant system during COVID-19. In support of this argument, a recent study showed that SARS-COV-2 leads to change in the intracellular/extracellular redox balance in the host immune cells, and the antioxidant defense was inhibited in the upper respiratory tract (Tavassolifar et al., 2023). Another study suggested that the cytokine storm in severe COVID-19 patients leads to a decrease in the antioxidant defense of endothelial cells through downregulation of the Nrf2 transcription factor (Rodrigues et al., 2023). Damaged mitochondria increase ROS and production of pro-inflammatory cytokine, along with the release of mtDNA leading to cell death, inflammation, and tissue damage. Taken together, all of these events can cause oxidative stress, hyperferritinemia, blood clotting, and thrombosis which are all reported in severe COVID-19 (Iessi et al., 2020).

### Mitochondrial metabolism

Mitochondria known for their vital role in cell bioenergy and metabolism, are central sites for metabolism including the TCA (Krebs) cycle, fatty acid β-oxidation (FAO), and OXPHOS (Garaude, 2019).

Studies have shown that many immune signaling pathways are highly integrated with cellular metabolism, which not only provides fuel for active cells but also provides guidance for deciding cellular fate. These studies have led to a new field of research called “immunometabolism”. Thus, mitochondrial metabolism is one of the important factors in innate and acquired immunity (Weinberg et al., 2015), since mitochondria can modulate metabolic and physiological states in different types of immune cells (de Souza Breda et al., 2019).

Viruses hijack host cell metabolism and cause alterations in cellular and physiological functions (Thaker et al., 2019). The function of central metabolic pathways, such as OXPHOS is often altered by viruses to provide energy, biosynthetic resources, or evade the immune system to facilitate their replication (Qu et al., 2019b). Reprogramming of various aspects of host central carbon metabolism has been reported by both DNA and RNA viruses, including increased glycolysis, and increased pentose phosphate activity to support nucleotide production, amino acid production, and lipid synthesis (Thaker et al., 2019). Also, since changes in amino acid metabolism are closely related to inflammatory and immune responses, amino acid metabolism can play a role in controlling pathogen infection and regulating inflammation. Arginine (Arg) has been proposed to be a key amino acid for viral replication of many DNA and RNA viruses (Tomé, 2021). Hijacking of host lipid metabolism by some viruses to improve viral replication has also been suggested (Gong et al., 2023).

Biochemical manifestations specific to SARS-CoV-2 infection include an acute inflammatory condition with cytokine storm and oxidative stress with devastating downstream effects such as chronic hypoxia, acidosis, hypercoagulation, and changes in aerobic glycolytic metabolism (Shenoy, 2020). Transcriptome data analysis showed an increase in the expression of genes associated with OXPHOS in both PBMC and in bronchoalveolar lavage fluid (BALF) of COVID-19 patients (Gardinassi et al., 2020). Ajaz et al. evaluated mitochondrial function in PBMC of COVID-19 patients. ATP-dependent respiration and maximal respiration represent a modified mitochondrial functional response to SARS-CoV-2 infection. This study confirmed mitochondrial dysfunction and metabolic alterations with increased glycolysis in PBMC of SARS-CoV-2 infected patients (Ajaz et al., 2021). Another study confirmed that the SARS-CoV-2 induces glycolysis and one-carbon metabolism and further supports viral RNA and protein expression, proliferation, and cytopathic effect (Zhang et al., 2021). SARS-CoV-2 spike protein subunits in human pulmonary microvascular endothelial cells showed changes in mitochondrial metabolism, indicating a change from aerobic to anaerobic metabolism (Zekri-Nechar et al., 2022). SARS-CoV-2 ORF-3c alters mitochondrial metabolism, causing a shift from glucose oxidation to fatty acids and increased oxidative phosphorylation (Mozzi et al., 2023). Also, COVID-19 patients have shown alterations in both lipid and amino acid metabolisms compared to healthy controls (Masoodi et al., 2022). Lipid metabolism is altered in COVID-19 patients through direct cellular infection as well as systemic inflammatory response. Elevated long-chain polyunsaturated fatty acid (PUFA) levels, in the absence of elevated long-chain acylcarnitines, which may reflect impaired mitochondrial fatty acid oxidation, suggest a possible role for phospholipase A2 in COVID-19 (Casari et al., 2021).

**Figure 2:**
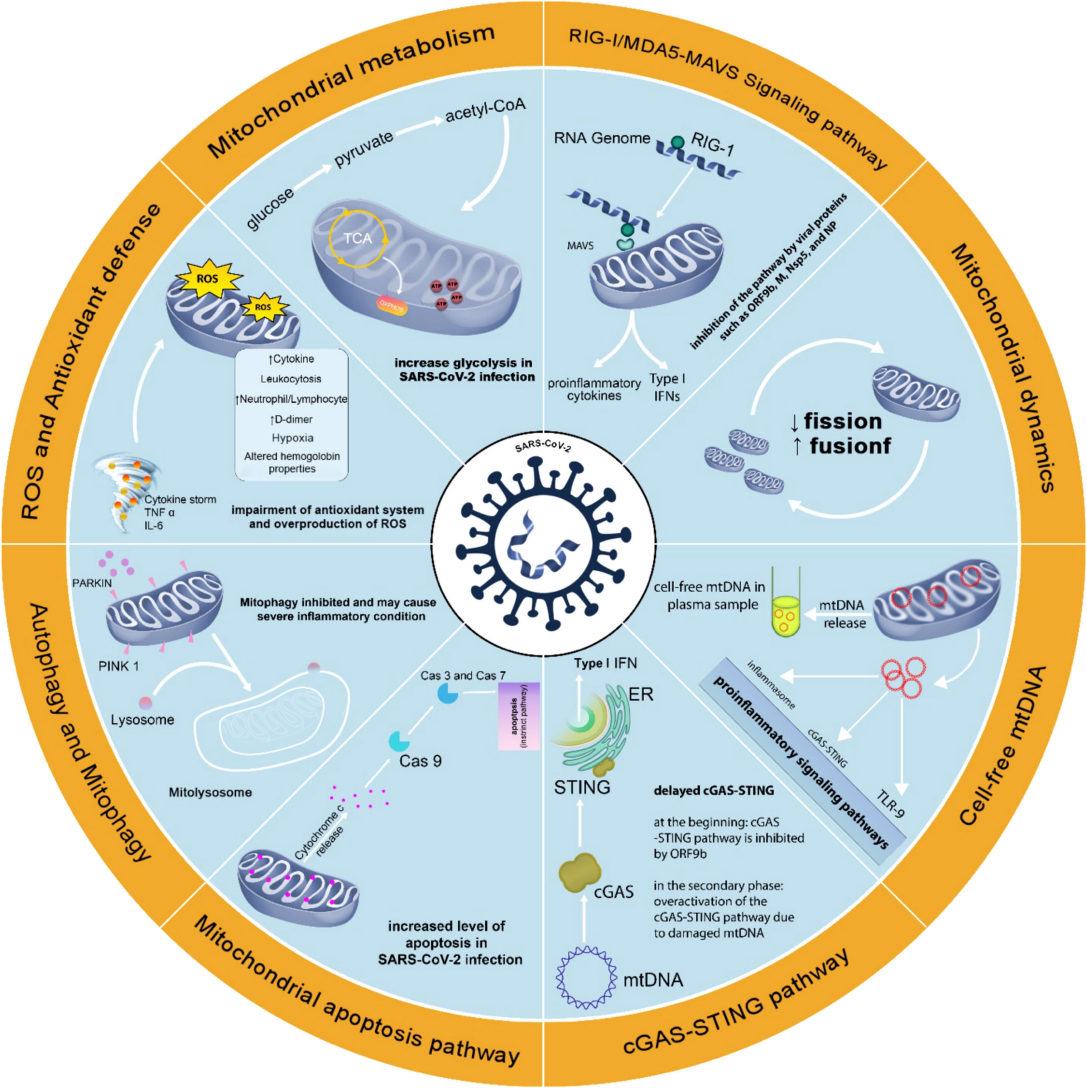
Changes in mitochondria-related events in SARS-CoV-2 infection. Changes in mitochondrial function in SARS-CoV-2 infection support the role of this organelle in COVID-19. Attention to mitochondrial function in SARS-CoV-2 infection may help better understand the pathophysiology of COVID-19 disease and achieve effective methods of prevention, diagnosis, and treatment.

Thus, the immune‐metabolic phenotype, including mitochondrial targeting could be used to understand the pathogenesis of the disease and possible treatments (Thompson et al., 2020). **Figure 2** summarizes the changes in mitochondria-related events in SARS-CoV-2 infection.

### Mitochondria-associated membranes

Mitochondria-associated membranes (MAMs), the points of contact between the ER and mitochondria, have been implicated in autophagy, apoptosis, mitochondrial dynamics, and activation of NLRP3 inflammasome (Namgaladze et al., 2019). In addition, recent studies have shown that some viruses exploit multiple ER membrane machineries to facilitate different stages of their life cycle (Woo et al., 2023). A 2023 study showed that hepatitis C virus replication requires the integrity of MAMs (Duponchel et al., 2023). A recent study by Jiao et al., showed that SARS-CoV-2 NSP6 induced ER stress which ultimately led to lysosomal degradation of STING and decreased IFN production (Jiao et al., 2023).

### Age-related mitochondrial dysfunction

There is a link between mitochondrial dysfunction and aging, age-related disorders, and metabolic syndromes. Age-related mitochondrial dysfunction could also be due to an imbalance of mitochondrial dynamics, autophagy and mitophagy, and biogenesis that result in the accumulation of damaged mitochondria (López-Lluch, 2017).

Immunosenescence has been described as age-related deterioration of the immune system. Accumulation of mitochondrial damage combined with reduction of the efficiency in energy production affects the capacity of the immune system through reducing the capacity to respond to viral infections by lower IFN-I release. Mitochondrial dysfunction releases damage signals to the cytosol, which leads to the release of inflammatory cytokines and the activation of the inflammasome, leading to chronic inflammation that is associated with aging and age-related diseases (Ayala et al., 2020).

Indeed, the decay of mitochondrial function is key to the progression of aging and age-related diseases. Mitochondrial dysfunction is also implicated in deteriorating immune systems as a key factor in inflammation, higher susceptibility to viral infections, and T cell immunodeficiency found in the elderly and age-related diseases (McGuire, 2019).

The COVID-19 severity is associated with impaired immune function found in the elderly, and more recently this aged immunity has been thought to intensify COVID-19. In addition, many age-related diseases such as metabolic syndromes, type 2 diabetes, obesity, hypertension, and cardiovascular disease exacerbate COVID-19 (Zheng et al., 2020). Age-related mitochondrial dynamic dysfunction has been reported in many studies. Therefore, the accumulation of inefficient mitochondria may be one of the causes of high COVID-19 mortality in the elderly, mainly due to mitochondrial dysfunction in senescent macrophages and lymphocytes that intensify the inflammatory response (Ayala et al., 2020).

Therefore, the study of mitochondrial immunity against SARS-CoV-2 could provide insight into why older people may have difficulty coping with COVID-19 (Ganji and Reddy, 2021).

### Cell-free mitochondrial DNA as a non-invasive biomarker

It is not unreasonable to expect that local and systemic changes following cell injury and disease are rapidly reflected in the cellular and molecular components of the blood (Hsiao and Hoppel, 2018). Because viruses can directly or indirectly modulate mitochondrial function in different cell types, the occurrence of mitochondrial alterations in blood cells could be a reflection of tissue mitochondrial changes (Cruz and Kang, 2018; Nunn et al., 2020).

Changes in somatic mtDNA have been considered as one of the primary aims in diagnosis and predicting various types of diseases, including infectious diseases (Qu et al., 2019a). One of the advantages of circulating mtDNA is the ability to be used as a liquid biopsy (González-Masiá et al., 2013). In recent years, mtDNA content has been considered a biomarker of mitochondrial function. mtDNA is also studied for disease-related changes in blood samples and other body fluids (Rosa et al., 2020). Among the advantages of mtDNA compared to nDNA are shorter length, easier and cheaper screening, high number of copies, and found in various fluids (Scozzi et al., 2021).

A recent study evaluated cf-mtDNA levels in the plasma of COVID-19 patients. Results showed plasma MT-CYTB levels had similar AUCs for predicting patient mortality compared with LDH, ferritin, and D-dimer. This study suggested high levels of circulating mtDNA as a poor biomarker for the prognosis of COVID-19 (Scozzi et al., 2021).

Although circulating mtDNA is one of the most common mtDAMPs evaluated as biomarker, other mitochondrial DAMPs may also be novel biomarker candidates (Shen et al., 2022). However, studies on mitochondria as a biomarker for the diagnosis or prognosis of COVID-19 are very limited.

### Mitochondrial targeted therapy for the treatment of COVID-19

**Figure 3:**
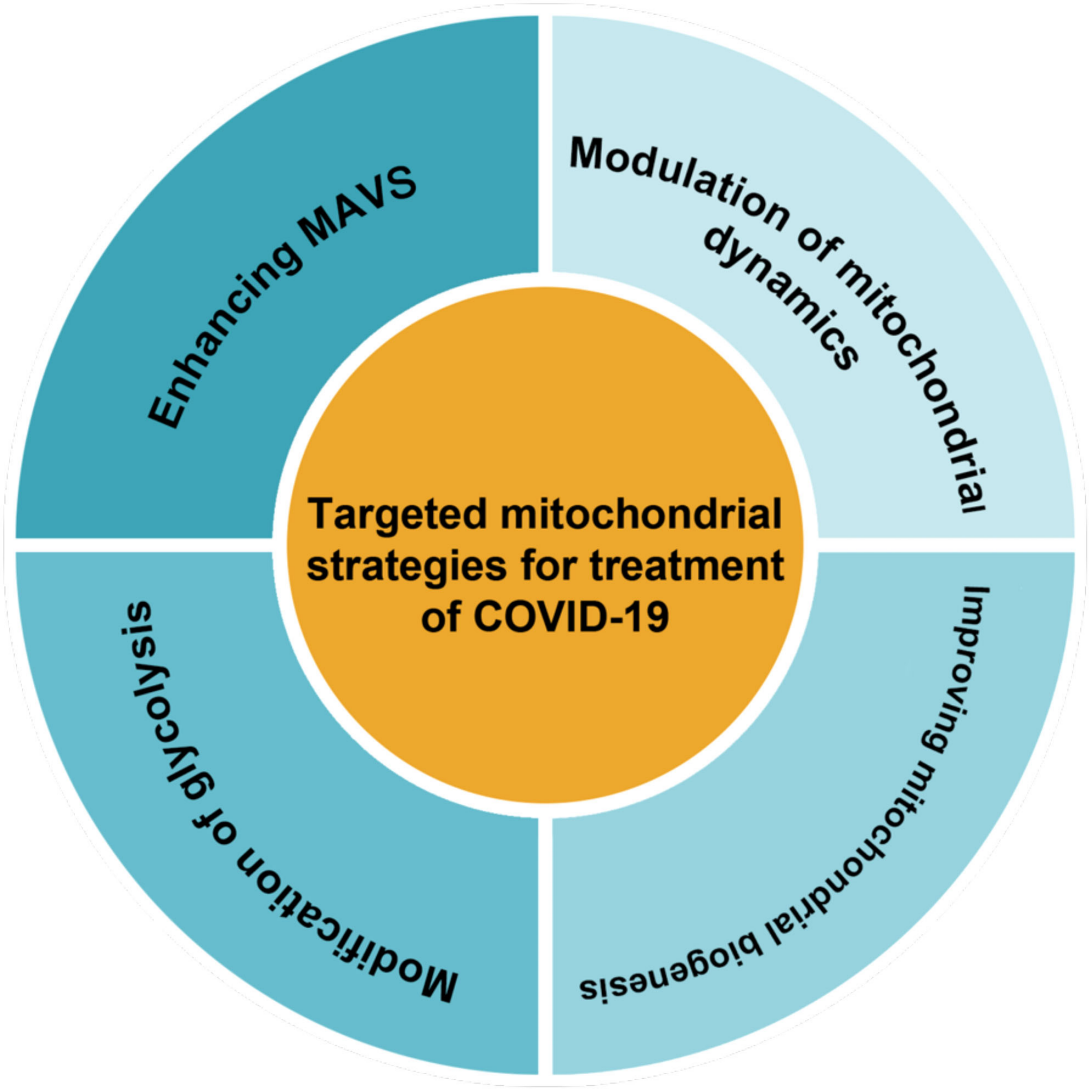
There are several methods for classifying targeted mitochondrial strategies.

Therapeutically targeting mitochondrial dysfunction might represent a successful strategy for disease (Thompson et al., 2020). As mitochondrial dysfunction has been shown to play an important role in inhibiting the antiviral response in infected cells. It seems that restoring mitochondrial function and creating new healthy mitochondria could enhance cellular resistance to infection-induced stress by regulating bioenergy and the innate immune response (Li et al., 2019). Therapies that improve mitochondrial function and inhibit inflammation may be some of the more effective treatments for COVID-19 (Holder and Reddy, 2021). Decreased inefficient mitochondria could diminish the inflammatory response to SARS-CoV-2 infection (Zheng et al., 2020). Also, a 2023 study reported that lower peripheral levels of mtDNA were associated with lower cellular immunity due to COVID-19 vaccination, suggesting that maintaining mitochondrial integrity is also essential for adequate immune responses to vaccination (Ikezaki et al., 2023). Possible therapeutic strategies for COVID-19 based on mitochondria as classified in **Figure 3** are reviewed in this section.

#### Modification of glycolysis

Metabolic phenotypes resulting from viral infection often reflect metabolic changes observed in cancer cells, such as positive regulation of nutrient intake and anabolism to support viral replication or rapid cell growth, respectively (Thaker et al., 2019). Recently targeted metabolic reprogramming of immune cells or cancerous cells in the treatment of various diseases such as cancers has been considered (Castro et al., 2019; Faubert et al., 2020). In addition, studies have been performed on targeted metabolic reprogramming to improve the effectiveness of viral therapy (Kennedy et al., 2020). The metabolic manipulation by SARS-CoV-2 stimulates an enhanced inflammatory response that helps symptoms of COVID-19. Targeting the mitochondrial metabolic pathways could help define new strategies for COVID-19 treatment (Ajaz et al., 2021). An enhanced glycolytic pathway upon SARS-CoV-2 infection is one example of viral infection-induced metabolic reprogramming. A study reported the use of a known glycolytic inhibitor, 2-deoxy-d-glucose (2-DG), to effectively counter SARS-CoV-2 replication in host cells (Bhatt et al., 2022).

#### Enhancing MAVS

Improving the MAVS pathway may be a possible treatment. Babajani et al. proposed a hypothesis for targeted therapy of COVID-19 with overexpressed MAVS protein from manipulated mesenchymal stem cells that express viral S protein to boost the innate immune response and the production of appropriate IFNs (Babajani et al., 2021).

#### Modulation of mitochondrial dynamics

A delay or absence of types I and III IFN responses with strong cytokine production was observed in COVID-19. Because there is a negative feedback loop between autophagy and the IFN-I response, inhibition or activation of autophagy is important depending on the stage of the SARS-CoV-2 infection. Inhibition of autophagy in the first stage of COVID-19 may lead to the regulation of the interferon antiviral response and inhibition of viral replication. Activation of autophagy in the late stage of the disease could lead to the removal of ROS, damaged organelles and reduce inflammation and balance the immune response. For these reasons, autophagy-related therapeutics should be selected according to the COVID-19 timeline (García-Pérez et al., 2020).

#### Improving mitochondrial biogenesis

Oxidative stress affects mitochondrial functions such as biogenesis and antioxidant defense mechanisms (Gao and Chen, 2022). The use of antioxidant compounds in SARS-CoV-2 infection is also recommended. These compounds scavenge ROS in a variety of ways and could therefore prevent the pathophysiological consequences of COVID-19 disease (Laforge et al., 2020; Camp et al., 2021). While ROS scavengers prevent the virus from entering the host cell, an effective dosage of ROS inducers could modify the reduction–oxidation (redox) potential in host cells to induce oxidative stress, resulting in the degradation of nascent RNA. It would largely affect viral RNA. Furthermore, the ROS causes cytotoxicity in infected cells as their DNA repair mechanism would be compromised (Nadhan et al., 2021). Oxidative stress and persistent pathogenesis in SARS-CoV-2 are almost certainly related. The cytokine storms and free radical storms should be considered in COVID-19 (Delgado-Roche and Mesta, 2020; Wu, 2020; de Oliveira et al., 2021).

### Mitochondria and long-COVID

Post-COVID-19 syndrome, or “long-COVID”, is a term that describes various conditions, such as inflammation and the consequences of organ damage that persist for a long time after SARS-CoV-2 infection. Although the mechanism underlying the long-COVID is not yet elucidated, the role of mitochondrial dysfunction and the subsequent immune response has recently received attention. It has been suggested that some post-COVID complications are related to mitochondrial dysfunction (Chen T-H. et al., 2023). The long-COVID status could be caused by damage related to the host response to initial severe infection, such as cytokine storms, and could lead to oxidative stress and oxidative and inflammatory damage. This suggests that antioxidant therapies may also be useful in the treatment of long-COVID (Wood et al., 2020; Chen T-H. et al., 2023).

## Conclusion

Manipulation of host mitochondria by SARS-CoV-2 plays a significant role in disease progression through modulation of various functions. The evidence indicates that SARS-CoV-2 infection could lead to modulation/disruption of the MAVS pathway, mitochondrial dynamics, apoptosis, autophagy and mitochondrial metabolism. Further research into the molecular mechanisms that hijack mitochondria in SARS-CoV-2 infection to suppress the immune system will provide insight into new treatment and prevention. Also, host mitochondria may be the key to effective and/or ineffective response in individuals, especially the elderly, to the COVID-19 vaccine. Improving or maintaining mitochondrial function may prevent dysfunction of mitochondria following viral infection. Targeted drug interventions that are effective in enhancing mitochondrial function may improve innate immunity against SARS-CoV-2 infection. In addition, evaluation of mitochondrial status and related markers in accessible tissues such as blood in response to SARS-CoV-2 may provide perspectives to predicting susceptibility to and the severity of COVID-19. High mtDNA copy number allows assessment of changes in different samples, which is also usually indicative of the mitochondrial biogenesis. Also, the small size of mtDNA provides an easy and cheap analysis method. However, the lack of studies on the role of other mitochondrial DAMPs in SARS-CoV-2 infection is noticeable.

## Author contributions

SRM: article decision, planning, and writing. SS: literature search and reviewing and article writing. AES: manuscript revision. AG: article reviewing. All authors contributed to the article and approved the submitted version.
